# New species of *Isotomiella* Bagnall, 1939 from Southeast of Brazil (Collembola, Isotomidae)

**DOI:** 10.3897/zookeys.233.3553

**Published:** 2012-10-26

**Authors:** Maria Cleide de Mendonça, Eduardo A. Abrantes, Ana Carolina R. Neves

**Affiliations:** 1 Departamento de Entomologia, Museu Nacional / Universidade Federal do Rio de Janeiro, Quinta da Boa Vista s/n, São Cristóvão, Rio de Janeiro, RJ 20940-040, Brazil

**Keywords:** Anurophorinae, Atlantic Rain Forest, Brazilian collembolan diversity, Taxonomy

## Abstract

Two new species of the genus *Isotomiella* Bagnall, 1939 are described and illustrated, the first: *Isotomiella macedoi* **sp. n.**, based on males and females, from the “Parque Nacional da Serra dos Órgãos” (Teresópolis municipality, State of Rio de Janeiro) differs from the other by tibiotarsus III thickened and blunt and two antero-lateral chaetae of labrum strongly thickened. The second species *Isotomiella uai* **sp. n.** from “Serra da Gandarela”, (Caeté municipality, State of Minas Gerais) differs from the other by presence of short sensilla on antennal IV and tergites, two anterolabral chaetae thickened and falcate mucro.

## Introduction

*Isotomiella* comprises so far a reduced number of species for a genus considerably diverse, especially in tropical and subtropical regions. This can be attributed to the aspect of its members, all white and small, that look at first sight identical. Nowadays, the genus comprises 49 species, of which 18 were described from Brazil (Deharveng and Oliveira 1990; Oliveira and Deharveng 1990; Mendonça and Fernandes 2003a, b; Mendonça and Abrantes 2007). Recent studies of the Isotomidae fauna in preserved areas of two States revealed two new species herein described and illustrated: *Isotomiella macedoi* sp. n. from “Parque Nacional da Serra dos Órgãos”, Teresópolis municipality, State of Rio de Janeiro, and *Isotomiella uai* sp. n. from “Serra da Gandarela,” Caeté municipality, State of Minas Gerais.

## Material and methods

The specimens studied were collected in litter and soil, extracted with Berlese-Tullgren funnels and mounted in glass slides according with the usual methodology. The type-material has been deposited in the Collembola Collection at the Departamento de Entomologia, Museu Nacional, Rio de Janeiro (CM/MNRJ).

### Abbreviations used in descriptions

Ant–antennal segment, Th–thoracic segment, Abd– abdominal segment, sl– lateral sensillum, sv– ventral sensillum, spl–dorsal lateral sensillum.

## Data resources

The data underpinning the analyses reported in this paper are deposited in the Dryad Data Repository at doi: 10.5061/dryad.m966q

## Taxonomy

### Isotomiella macedoi sp. n.

urn:lsid:zoobank.org:act:48E60458-A1F8-4C63-9C6B-28CA3F6A8724

http://species-id.net/wiki/Isotomiella_macedoi

Figs 1 13

#### Material.

Holotype: Male (2320 CM/MNRJ): Southeast Brazil, State of Rio de Janeiro, Teresópolis municipality, Rain forest litter at 1.400 meters a.s.l., 14-III-2012. Mendonça, M.C. leg. One Paratype male, same data as holotype, One male and 12 Paratypes female (2364CM/MNRJ) Same locality of holotype. 31-V-2012. All material deposited in Collembola Collection of Museu Nacional da Universidade Federal do Rio de Janeiro (Brazil).

#### Description.

Total body length of the holotype 0,74 mm. Habitus typical of the genus. Color white. Integument without craters. Pseudopores and integument channels not observed.

**Figure 1–6.**
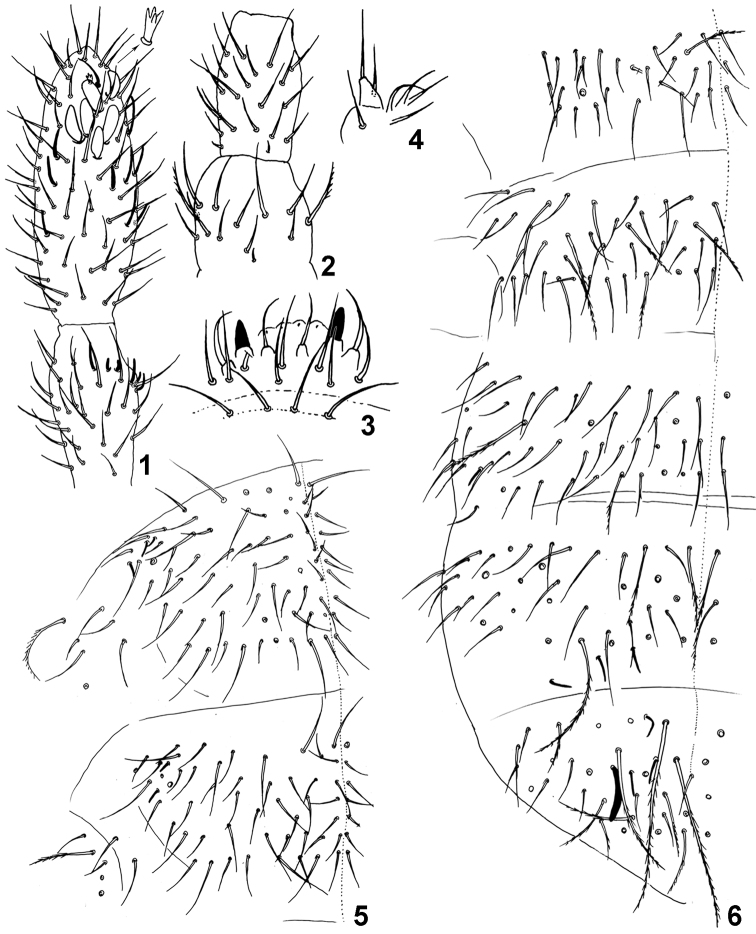
*Isotomiella macedoi* sp.n. **1** Ant III-IV Dorsal view, detail of the apical microsensillum **2** Ant I-II Dorsal view **3** Labral and prelabral chaetae **4** Outer lobe of maxilla **5** Dorsal chaetotaxy of Th II-III **6** Dorsal chaetotaxy of Abd I-VI.

**Figure 7–13.**
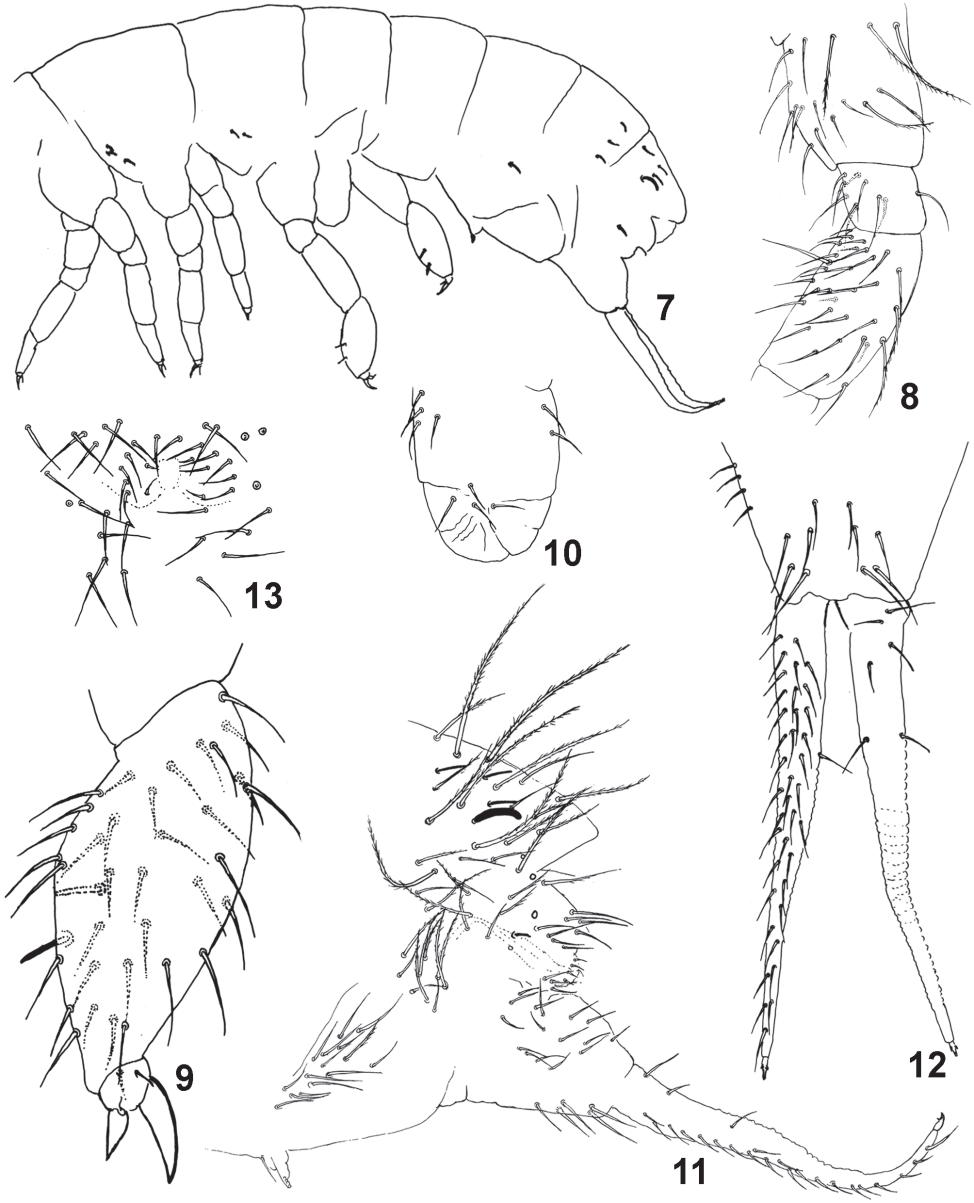
*Isotomiella macedoi* sp.n. **7** Sensillary pattern of the body **8** Subcoxa and femur of leg III **9** Tibiotarsus and unguis of leg III **10** Lateral view of ventral tube **11** Lateral view of abd. V-VI, subcoxa furcal, furca and tenaculum **12** Furca **13** Male genital plate.

Antennae subequal to head diagonal. Length of Ant I to IV (30 µm, 40 µm, 40 µm, 65 µm). Ant IV with one conspicuous tulip-shaped microsensillum with three or four small spines at apex protected by a curved chaeta, six broad and subequal sensilla, eight supplementary very thin sensilla (seven dorsal external, six dorsal internal and one shorter and thicker dorsal). Ant III with about 35-40 ordinary chaetae, two small sensory rods (3 µm), three guard sensilla (7 µm) and one short sensillum below (Fig. 1). Ant II with about 40 smooth ordinary chaetae, 2 basal microsensilla, one dorsal and one ventral, and one dorso-lateral sensillum (Fig. 2). Ant I with 14 smooth ordinary chaetae, two lateral ciliated chaetae, two ventral and unequal sensilla (10 µm and 4 µm) and two basal microsensilla, one dorsal and one ventral. Labral chaetae pattern 4/5,5,4, the third row with two lateral acuminate chaetae and the last row with two anterolateral chaetae strongly thickened (Fig. 3). External lobe of maxilla with bifurcate palp and four sublobal chaetae (Fig. 4). All chaetae of head smooth. Axial chaetotaxy pattern from Th II to Abd IV as 20,14/6,6,6,6 by half tergite. Th II with 1+1 lateral ciliated macrochaetae (45 µm) and 3+3 lateral sensilla (5 µm). Th III with 1+1 lateral ciliated macrochaetae (50 µm) and 2+2 lateral sensilla (5 µm); all the other chaetae smooth (Fig. 5). Abd I with 1+1 lateral ciliated macrochaetae (40 µm) and 2+2 ciliated chaetae (30 µm) between the lateral macrochaetae; sensilla absent. Abd II with 1+1 lateral ciliated macrochaetae (40 µm) and 2+2 ciliated chaetae between lateral macrochaetae; sensilla absent and without the lateral area devoid of chaetae. Abd III with 2+2 anterior ciliated chaetae (35 µm) and 2+2 posterior ciliated chaetae (35 µm ) between the lateral ciliated macrochaetae (40 µm), 1+1 ventral sensillum (5 µm). Abd IV with many ciliated chaetae (35-50 µm) and few smooth chaetae, 3+3 posterior sensilla (11 µm). Abd V-VI with several ciliated macrochaetae of different sizes (60-75 µm) and few smooth chaetae, 1+1 long and thick dorso-lateral sensillum (**spl)** with apex slightly curved (20 µm), 3+3 dorso-lateral sensilla **sa**, **spe**, **spi** (11 µm) and 1+1 ventral sensillum **sv** (5 µm) (Fig. 6). Dorso-lateral sensillary pattern of the body 3,2/0,0,1,2,5 (Fig. 7) Subcoxa I of legs I, II, III with 2, 3, 2 ciliated chaetae. Femur III with tenent hair smooth and two ciliated chaetae (Fig. 8). Tibiotarsus III strongly thickened on males, with about 40 smooth chaetae and 2 thin erect stick-like chaetae (10 µm); on females the tibiotarsus III are normal without stick-like chaetae. Unguis plump (20 µm) and unguiculus (10 µm) lanceolated (Fig. 9). Ventral tube (45 µm) with 4+4 anterior, 2+2 posterior and 4+4 distal chaetae (Fig. 10). Tenaculum with 4+4 teeth and 1 chaeta. Subcoxae anterior with 15 chaetae of which one ciliated; subcoxae posterior with 9 chaetae of which 6 ciliated (Fig. 11). Manubrium (55 µm) with 5+5 ventro-distal, 4+4 lateral, and 12+12 dorsal smooth chaetae. Dens long, (125-130 µm) crenulated, with about 40 anterior and 6 posterior smooth chaetae. Mucro small (7 µm), tridentate, the 2 basal teeth symmetrical (Fig. 11-12). Male genital plate as in Fig. 13.

#### Remarks.

*Isotomiella macedoi* sp. n. is easily included in the *minor* group *sensu* Kovác and Palacios-Vargas (2008) by its sensillar pattern (3,2/0,0,1,3,5), 3+3 to 5+5 ventral chaetae of manubrium, mucro tridentate and 4 sublobal chaetae. However, the new species also shares some characters, which do not define groups, with species of the *nummulifer* groups. With *Isotomiella delamarei* Barra, 1968, *Isotomiella spinifer* Deharveng & Oliveira, 1990, *Isotomiella edaphica* Bedos & Deharveng, 1994, *Isotomiella leksawasdii* Bedos & Deharveng, 1994, *Isotomiella canina* Mendonça & Fernandes, 2003, the new species shares the antero-lateral chaetae of labrum strongly thickened and blunt. With *Isotomiella digitata* Deharveng & Oliveira, 1990,* I. distincta* Mendonça & Fernandes, 2003 and *Isotomiella falcata* Mendonça & Fernandes, 2003, it shares the tibiotarsus III thickened. However the presence of a set of rare characters, in particular the tibiotarsus III strongly thickened with two thin erected stick-like chaetae in the males makes *Isotomiella macedoi* sp. n. unique among the genus *Isotomiella*. Females exhibit same characters as males, except this modified tibiotarsus III and stick-like chaetae.

#### Name derivation.

The species is dedicated to the husband of the senior author, Prof. Antonio Carlos M. Macedo, micropaleontologist.

### Isotomiella uai sp. n.

urn:lsid:zoobank.org:act:7DE12EBF-102F-4604-B46F-6C81C7688F11

http://species-id.net/wiki/Isotomiella_uai

Figs 14 25

#### Material.

Holotype: Female (2109CM/MNRJ), Southeast Brazil, State of Minas Gerais, Caeté municipality, Rain forest litter with about 1.500 meters a.s.l., 09. VII. 2011, Abrantes, EA and Silveira, TC leg.

Paratype: five females (2109CM/MNRJ) and six females (2110CM/MNRJ) same data as holotype, all material deposited in Collembola Collection of Museu Nacional da Universidade Federal do Rio de Janeiro, (Brazil).

#### Description.

**Figure 14–18.**
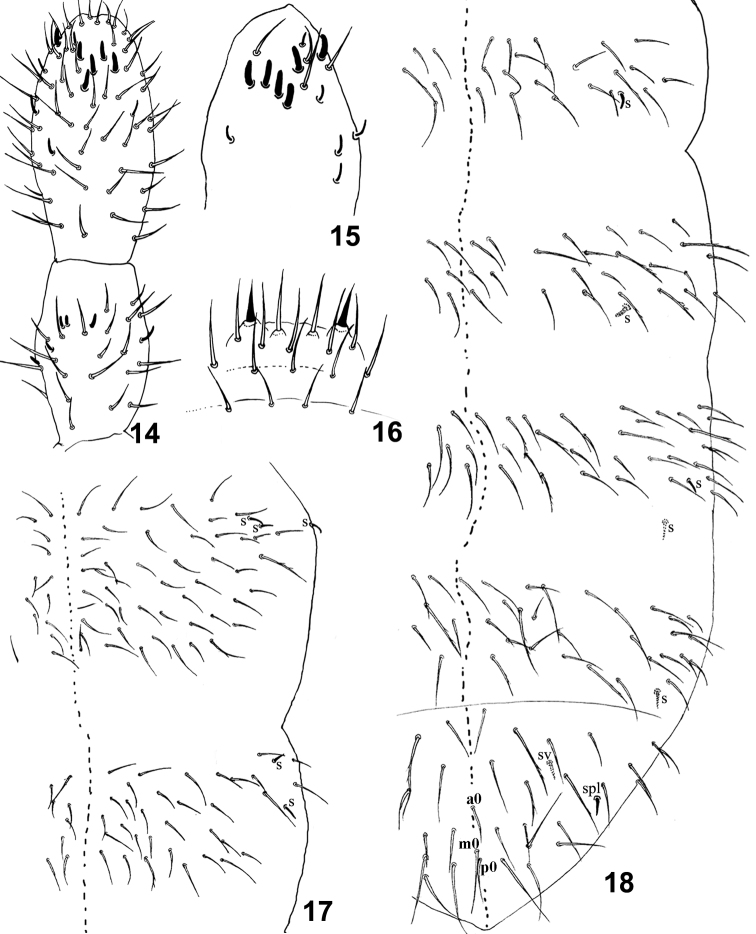
*Isotomiella uai*sp.n. **14** Ant III-IV Dorsal view **15** Sensillary pattern of Ant IV **16** Labral chaetae **17** Dorsal chaetotaxy of Th II-III **18** Dorsal chaetotaxy of Abd I-VI.

**Figure 19–25.**
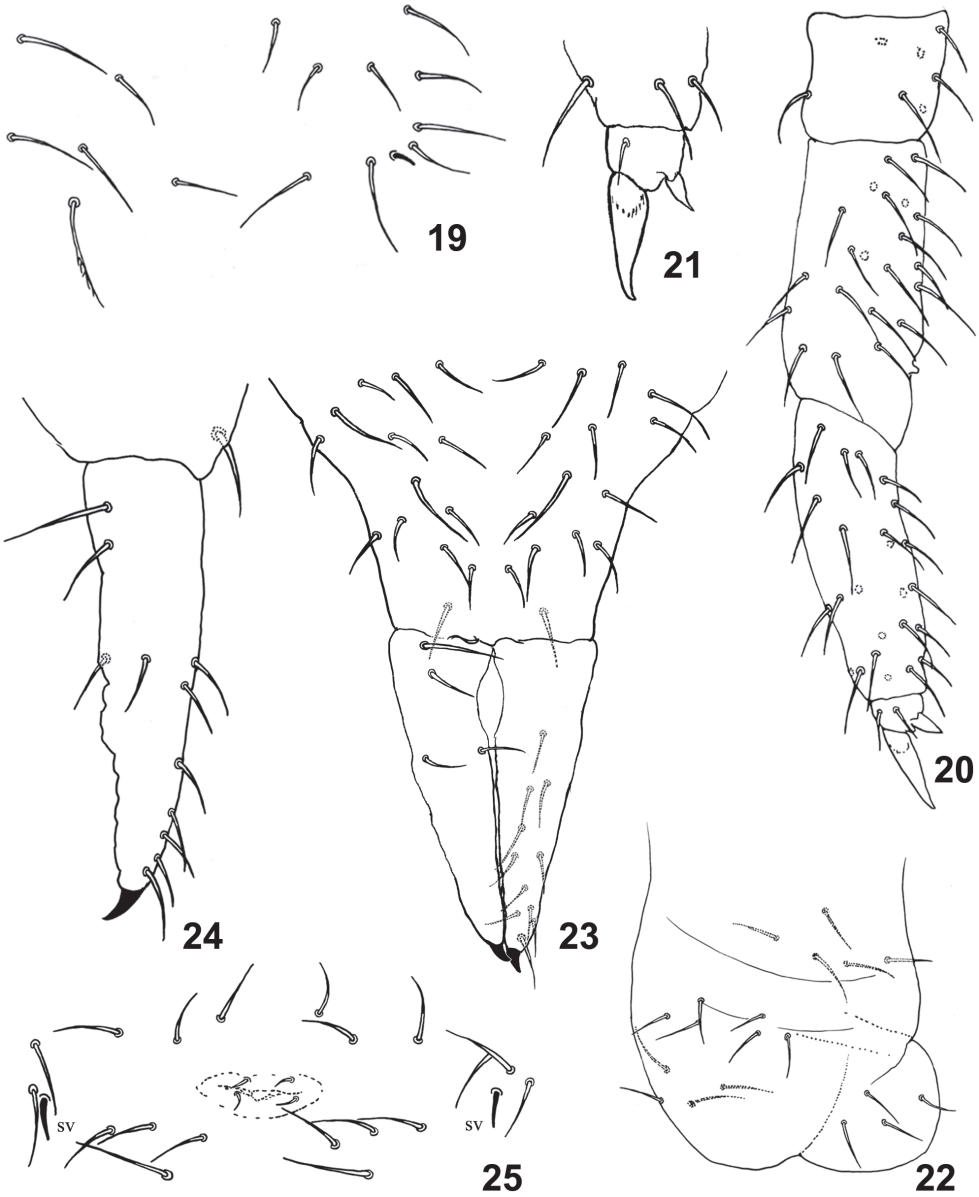
*Isotomiella uai* sp.n. **19** Detail of chaetotaxy of Abd II **20** Leg III **21** Unguis of leg III **22** Ventral tube **23** Furca **24** Lateral view of dens and mucro **25** Female genital opening.

Body length size 0,77–0,97mm. Habitus typical of the genus. Color white. Integument dorsally without craters, with primary granules. Pseudopores and integument channels not observed. Antennae subequal to head diagonal. Length of Ant I to IV (30 µm, 35 µm, 39 µm, 58 µm). Ant IV with one microsensillum protected by a curved chaeta, six small and subequal sensilla (5 µm), supplementary very thin sensilla (3-4 dorsal lateral external and one dorsal internal) (Figs 14-15). Ant III with 26 ordinary chaetae, two very small sensory rods (2 µm), three small guard sensilla (3 µm), one below short sensillum and one lateral internal additional sensillum (Fig. 14). Ant II with 23-24 smooth ordinary chaetae, two basal microsensilla (one dorsal lateral and one ventral lateral) and one microsensillum lateral external. Ant I with about 13 dorsal ordinary chaetae, two ventral and unequal sensilla (5 µm and 3 µm) and two basal microsensilla (one ventral and one dorsal). Labral chaetae pattern 4/5,5,4, four very little anterior spinules; two antero lateral chaetae of labrum (11 µm) thickened with apical filament (Fig. 16). External lobe of maxilla with bifurcate palp and 3 sublobal chaetae. All chaetae of head smooth. Axial chaetotaxy pattern from Th II to Abd IV with 8-10,5/3,3,3,3 by half tergite (Figs 17, 18). Th II with 1+1 lateral macrochaetae slightly ciliated (22 µm) and 3+3 lateral sensilla, **sl 3** (5 µm) migrated far from the others two (5 µm and 3 µm); Th III with 1+1 lateral slightly ciliated macrochaetae (22 µm) and 2+2 lateral sensilla (5 µm) (Fig. 17). Abd I with 1+1 lateral slightly ciliated chaetae (17 µm) and 1+1 sensillum (4 µm); Abd II with 1+1 lateral slightly ciliated macrochaetae (22 µm), 1+1 ventral-lateral sensillum, without area devoid of chaetae (Figs 18, 19). Abd III with 1+1 slightly ciliated macrochaetae (22 µm), few slightly ciliated chaetae (18 µm) and 2+2 sensilla (5 µm), one dorso-lateral and one ventral. Abd IV with 2+2 ciliated macrochaetae (23 µm) and 1+1 ventral-lateral sensilla (7 µm). Abd V-VI with some slightly ciliated (30 µm) and some smooth macrochaetae; 1+1 dorso-lateral sensillum **spl,** short and thin (5 µm); 1+1 ventral sensillum **sv** (6 µm) and unpaired chaetae smooth, **ao** (15 µm), **mo** (19 µm) and **po** (20 µm) (Fig. 18).

Proximal whorl of tibiotarsi with seven chaetae (Fig. 20). Tibiotarsus III without tenent hair or thickened apical chaetae. Unguis thin and toothless (15 µm), unguiculus lanceolate (5 µm) (Figs 20, 21). Ventral tube with 3+3 anterior, 4-5 posterior and 4+4 distal chaetae (Fig. 22). Tenaculum with 4+4 teeth and one chaeta. Subcoxae anterior with seven chaetae of which one ciliated; subcoxae posterior with seven chaetae, of which one ciliated. Manubrium (55 µm) with 1+1 ventro-distal and 14+14 dorsal chaetae, without lateral chaetae (Fig. 23). Dens longer than manubrium (65 µm), slightly crenulated, with 10+10 – 13+13 anterior and 4+4 posterior chaetae. Mucro small and falcate (5 µm) (Fig. 23, 24). Female genital plate as in Figure 25.

#### Remarks.

*Isotomiella uai* sp. n.belongs to the *nummulifer* group *sensu* Kovác and Palacios-Vargas (2008) that is defined by sensillary pattern 3,2/1,1,2,1,2 from Th II to Abd IV, reduced body chaetotaxy, manubrium usually with 1+1 to 2+2 ventral chaeta, mucro usually bidentate or falcate mucro and 3 external sublobal chaetae. This new species is closest to *Isotomiella fellina* Mendonça & Fernandes, 2003 by its reduced general chaetotaxy and short sensilla on both antennae and tergites, but differs from it by the 1+1 ventral chaetae on manubrium, 10-13 ventral and 4 dorsal chaetae on dens and falcate mucro, against 2+2 ventral chaetae on manubrium, 19-21 ventral and 5 dorsal chaetae on dens and bidentate mucro in *Isotomiella fellina*. The falcate mucro of the new species is also similar to that of *Isotomiella barrai* Deharveng & Oliveira, 1990, *Isotomiella annae* Deharveng & Suhardjorno, 1994 and *Isotomiella falcata* Mendonça & Fernandes, 2003 and *Isotomiella proxima* Mendonça & Fernandes, 2003, but other characters are quite distincts. Therefore, *Isotomiella uai* sp.n. differs from all species of the genus by a singular set of characters, including short sensilla on both antennal IV and tergites, two anterolabral chaetae of labrum thickened and acuminate and falcate mucro.

#### Name derivation.

The species is dedicated to the people of Minas Gerais by the unique and local interjection **“uai”** used by its inhabitants.

## Supplementary Material

XML Treatment for Isotomiella macedoi

XML Treatment for Isotomiella uai
